# StratosPHere 2: study protocol for a response-adaptive randomised placebo-controlled phase II trial to evaluate hydroxychloroquine and phenylbutyrate in pulmonary arterial hypertension caused by mutations in BMPR2

**DOI:** 10.1186/s13063-024-08485-z

**Published:** 2024-10-15

**Authors:** Nina Deliu, Rajenki Das, Angelique May, Joseph Newman, Jo Steele, Melissa Duckworth, Rowena J. Jones, Martin R. Wilkins, Mark R. Toshner, Sofia S. Villar

**Affiliations:** 1 MRC Biostatistics Unit, Cambridge University, Cambridge, UK; 2 MEMOTEF, Sapienza University, Rome, Italy; 3 VPD-HLRI, Department of Medicine, University of Cambridge, Cambridge, UK; 4 Royal Papworth Hospital, Cambridge, UK; 5 Papworth Trials Unit Collaboration, Royal Papworth Hospital, Cambridge, UK; 6 Imperial College London, Heart Lung Research Institute, London, UK

**Keywords:** Pulmonary arterial hypertension, BMPR2 mutations, Adaptive design, Phase II trial, Bayesian response-adaptive randomisation, Precision medicine

## Abstract

**Background:**

Pulmonary arterial hypertension is a life-threatening progressive disorder characterised by high blood pressure (hypertension) in the arteries of the lungs (pulmonary artery). Although treatable, there is no known cure for this rare disorder, and its exact cause is unknown. Mutations in the bone morphogenetic protein receptor type-2 (BMPR2) are the most common genetic cause of familial pulmonary arterial hypertension. This study represents the first-ever trial of treatments aimed at directly rescuing the BMPR2 pathway, repurposing two drugs that have shown promise at restoring levels of BMPR2 signalling: hydroxychloroquine and phenylbutyrate.

**Methods:**

This three-armed phase II precision medicine study will investigate BMPR2 target engagement and explore the efficacy of two repurposed therapies in pulmonary arterial hypertension patients with BMPR2 mutations. Patients will be stratified based on two BMPR2 mutation classes: missense and haploinsufficient mutations. Eligible subjects will be randomised to one of the three arms (two active therapy arms and a placebo arm, all plus standard of care) following a Bayesian response-adaptive design implemented independently in each stratum and updated in response to a novel panel of primary biomarkers designed to assess biological modification of the disease.

**Discussion:**

The results of this trial will provide the first randomised evidence of the efficacy of these therapies to rescue BMPR2 function and will efficiently explore the potential for a differential response of these therapies per mutation class to address causes rather than consequences of this rare disease.

**Trial registration:**

The study has been registered with ISRCTN (ISRCTN10304915, 22/09/2023).

## Administrative information

Note: the numbers in curly brackets in this protocol refer to SPIRIT checklist item numbers. The order of the items has been modified to group similar items (see http://www.equator-network.org/reporting-guidelines/spirit-2013-statement-defining-standard-protocol-items-for-clinical-trials/).

**Table:** 

Title {1}	StratosPHere 2: study protocol for a response-adaptive randomised placebo-controlled phase II trial to evaluate hydroxychloroquine and phenylbutyrate in pulmonary arterial hypertension caused by mutations in BMPR2
Trial registration {2a and 2b}	ISRCTN: Trial ID ISRCTN10304915, dated 22/09/2023
Protocol version {3}	Protocol version number 2.0 dated 15/08/2023
Funding {4}	Medical Research Council (MRC) Development Funding Pathway Scheme (DPFS) Award MR/T025646/1
Author details {5a}	Nina Deliu, Lead Blinded Statistician (MRC Biostatistics Unit, Cambridge University; MEMOTEF, Sapienza University) Rajenki Das, Lead Unblinded Statistician (MRC Biostatistics Unit, Cambridge University) Angelique May, Clinical Trials Manager (VPD-HLRI, University of Cambridge Department of Medicine) Joseph Newman, Clinical Research Fellow (VPD-HLRI, University of Cambridge Department of Medicine; Royal Papworth Hospital) Jo Steele, Senior Clinical Trials Data Manager (Papworth Trials Unit Collaboration, Royal Papworth Hospital) Melissa Duckworth, Clinical Project Manager (Papworth Trials Unit Collaboration, Royal Papworth Hospital) Rowena J. Jones, Research Scientist (VPD-HLRI, University of Cambridge Department of Medicine) Martin R. Wilkins, Co-Investigator (Imperial College London, Heart Lung Research Institute) Mark R. Toshner, Principal Investigator (VPD-HLRI, University of Cambridge Department of Medicine; Royal Papworth Hospital) Sofia S. Villar, Senior Lead Blinded Statistician and Co-Investigator (MRC Biostatistics Unit, Cambridge University)
Name and contact information for the trial sponsor {5b}	Royal Papworth Hospital NHS Foundation Trust, Papworth Road, Cambridge Biomedical Campus, Cambridge, CB2 0AY Tel: 01223 638,000 Website: https://royalpapworth.nhs.uk/research-and-development
Role of sponsor {5c}	Papworth Trials Unit Collaboration (PTUC), a fully accredited UKCRC Clinical Trials Unit, will oversee the study and provide project management oversight, trial management, data management, statistical and research governance support as well as input into the overall study design and statistical methodology. PTUC also hold overall authority over publications in line with funder.

## Introduction

### Background and rationale {6a}

Pulmonary arterial hypertension (PAH) is a life-threatening disease where progressive narrowing of the pulmonary arteries leads to heart failure and, if untreated, premature death. Patients are typically diagnosed young and face life-long invasive and costly treatments including continuous intravenous infusions and eventually lung transplantation. There is no current cure. According to the 2020–2021 NHS National Audit of Pulmonary Hypertension Report, in the UK, the 5-year survival from diagnosis is 51% for idiopathic, heritable or drug-induced PAH without comorbidities (NHS [1]). Currently, available therapies treat the consequences, not the underlying cause of PAH. The costs of this approach are high. A recent pharmacoeconomic evaluation demonstrated oral therapy, discounted-QALY costs ranging from C$146,254–C$412,979 [2]. Though costs for oral therapy are coming down with generic availability, this is not the case for IV therapy and previous economic analyses are still valid with the cost-effectiveness ratio at £343,000/QALY [3]. This does not factor in the burden of transplantation, a common end-destination therapy, both economic and personal. Mutations in the transforming growth factor beta (TGFβ) family member, bone morphogenetic protein type-2 receptor type-2 (BMPR2) are causal in ~ 75% familial cases and 11–40% idiopathic PAH [4]. There are currently no therapies targeting the BMPR2 pathway, though, recent evidence from a phase III trial of the TGFβ-ligand trap (sotatercept) has shown a statistically significant effect of the experimental group compared with the placebo group, increasing the 6-min walk distance by 40.8 m (95% CI, 27.5–54.1, *p* < 0.001) from baseline to week 24 [5], therefore, indicating that modulation of the TGFβ pathway shows beneficial impact upon disease.

Different mutations affect BMPR2 function and expression in potentially complex ways that may lead to differential effects of treatment options. StratosPHere 2 addresses this by using a precision medicine framework that stratifies the trial by partitioning patient cohorts into strata defined by the BMPR2 mutation type. The rationale for the proposed approach is that the underlying cause of BMPR2-PAH is reduced BMPR2 pathway signalling. Variation in functional consequences of differing BMPR2 mutations suggests that approaches that target particular classes of mutation are applicable. In preclinical models, functional rescue of specific types of mutations can be achieved. This has included prevention of lysosomal degradation of the protein with hydroxychloroquine [6, 7] and improving endoplasmic reticulum (ER) stress and transit with phenylbutyrate [8].

Phenylbutyrate exerts effects through targeting both ER stress and misfolding. Missense mutations demonstrate retention of misfolded BMPR2, exerting a dominant-negative effect partly mediated by impaired trafficking of the associated type-I receptor [9]. Phenylbutyrate has been demonstrated to have independent effects on ER stress and inhibition of histone deacetylase [10], both therapeutic targets in PAH and therefore of potential relevance outside of BMPR2-PAH. Phenylbutyrate prevented and reversed PAH in disease in 2 distinct murine models of PAH-chronic hypoxia–induced and monocrotaline models [11]. BMPR2 turnover is by lysosomal degradation [12]. Hydroxychloroquine/chloroquine prevents lysosomal degradation, rescues BMPR2 signalling and attenuates PAH in preclinical models [6, 7]. Hydroxychloroquine does not affect ER folding or retention and therefore may have limited effects in the context of dominant negative effects of ER trafficking in missense mutations. Hydroxychloroquine is hypothesised to have the clearest effect in haploinsufficiency by increasing wild-type, normally functioning BMPR2, though wild-type rescue may still be therapeutic in missense mutations.

Currently, PAH remains a life-limiting disease with a profound quality of life and symptom burden. This study proposes the first-ever trial of treatments that target the genetic BMPR2 pathway of the disease, with the potential development of two repurposed drugs, in addition to the substantial benefit to PAH patients. Both therapies would be easier to administer than continuous IV infusions which also require the additional training of carers to administer and involve heavy healthcare utilisation. This study also fits into the current paradigm of precision medicine trials, by investigating the potential for personalising therapy recommendations according to the mutation class. Modification of the disease pathology will be measured using a novel panel of biomarkers to assess therapeutic effect on downstream transcriptomic signals of the BMPR2 pathway as a measure of BMPR2 pathway target engagement. To this end, the StratosPHere 1 trial, a prospective biomarker trial, was designed to identify biological biomarkers representative of the BMPR2 pathway (Jones RJ, De Bie EM.D.D., Ng AYK, Dunmore BJ, Deliu N, Graf S, Lawrie A, Newman J, Polwarth G, Rhodes C, Hemnes A, West J, Villar SS, Upton PD, UK National Cohort Study of Idiopathic and Heritable PAH Consortium, the Uniphy Clinical Trials Network and Toshner MR: BMPR-II biomarkers for testing therapeutic efficacy in pulmonary arterial hypertension – novel findings from the StratosPHere 1 study, 2024+, under review).

## Objectives {7}

### Primary objective

The primary objective is to determine if any of the two repurposed treatments can achieve target engagement in the two PAH strata of interest. Target engagement is defined by the demonstration of BMPR2 function using a panel of quantitative PCR (qPCR) measurements of BMPR2 target genes in peripheral blood. Target engagement will be quantified in terms of a combination of the individual changes in 8 genes from baseline (study entry; prior to any treatment) to follow-up (8 weeks from treatment initiation) using quantitative qPCR methodology performed independently for each of the two mutation strata and for each of the treatments considered. The 8-week follow-up timeframe for the primary endpoint is dictated by the pharmacokinetics aspects of hydroxychloroquine, which has a half-life of 30–50 days.

### Secondary objectives

- To evaluate the effectiveness of any of the two repurposed therapies in the pooled trial sample (independently on the stratum) by 6-minute walk test (6MWT) functional assessment and cardiac functional assessment (NT-proBNP)
- To assess the efficacy as defined by measures relevant to patient-related outcomes and function (EMPHASIS-10)
- To explore additional secondary measurements of BMPR2 expression and function (including different follow-up times and different normalisation methods; see Section 12). This would inform the definition of primary and secondary outcomes for studies exploring similar hypotheses regarding target engagement of BMPR2 function.

## Trial design {8}

StratosPHere 2 is a placebo-controlled (veiled as described in Senn, 1995 [13]), three-armed response-adaptive randomised controlled trial of two active arms (T1 = hydroxychloroquine + SoC; T2 = phenylbutyrate + SoC) and a control group (C = placebo + SoC). See Table 1 for more details. As the action mechanism of the two active arms differs in mutation subtypes, the enrolled population is stratified into two mutation strata, and the response-adaptive arm allocations are implemented and updated independently for each mutation class stratum.

**Table 1:** Study arms and dosing

	Treatment	Dosing	Body weight
*Arm 1 (T1)*	Hydroxychloroquine tablets + SoC	200 mg OD or BD oral tablets	Ideal^a^ not actual
		Max of 6.5 mg/kg/day with doses between 200 and 400 mg rounded down to 200 mg once daily	
*Arm 2 (T2)*	Glycerol phenylbutyrate liquid + SoC	1.1 g/ml oral liquid (25 ml bottles)	
		7 ml/m^2^/day	BSA^b^ ≥ 1.3 m^2^
		8.5 ml/m^2^/day	BSA < 1.3 m^2^
*Placebo control* + *SoC (C1/C2)*	Placebo tablets + SoC (C1)	200 mg OD or BD oral tablets	Ideal not actual
		Max of 6.5 mg/kg/day with doses between 200 and 400 mg rounded down to 200 mg once daily	
	Placebo liquid + SoC (C2)	1.1 g/ml oral liquid (25 ml bottles)	
		7 ml/m^2^/day	BSA ≥ 1.3 m^2^
		8.5 ml/m^2^/day	BSA < 1.3 m^2^

^a^Devine formula

^b^Du Bois method

### Adaptive allocation scheme

For each independent stratum, 20 patients are expected to be enrolled according to the following 3-stage adaptive design, whose details (e.g. number of blocks and individuals in each block) were chosen by extensive simulations to optimise the design’s operational characteristics. Supporting simulation studies will be provided in the statistical analysis plan (SAP).

- Stage 1: An initial block of 6 patients is expected to be enrolled and randomised to treatment arms based on a 2:2:2 allocation ratio (C:T1:T2).
- Stage 2: Once all patients from the first block (stage 1) of a given stratum will have had an observed response at the 8-week follow-up, the first interim analyses will inform the allocation probabilities of the second block of 6 targeted patients (stage 2) of the same stratum. This will be computed according to a Bayesian algorithm (described below), with the restriction that no arm can be dropped at this second stage.
- Stage 3: A second interim analysis will guide the update of the allocation probabilities of the third (and final) block targeting 8 patients in each stratum. The adaptation will account for stage 2 as well as stage 1 response data of each independent stratum. Futile active arms but not the control arms will be allowed to be dropped at this final stage. The futility or dropping criterion will be dictated by the Bayesian response-adaptive algorithm as a function of the allocation probabilities where the critical allocation probability threshold for dropping an arm is pre-determined and fixed. Its value is documented internally and will be publicly disclosed at the end of the study to minimise the level of information those who enrol participants or assign interventions will have about the algorithm’s recommended allocations.

### Bayesian response-adaptive algorithm

This response-adaptive trial follows the Bayesian design proposed in Trippa et al. [14] and Wason and Trippa [15] and starts with weakly-informative equal priors for all arms, that is, beta distributions ${Beta(\alpha }_{k}=1,{\beta }_{k}=1)$, for arms $k = C,\,T1,\,T2$. The limited informativity in the priors has been chosen to reflect that this is a first trial of its kind, and that no historical knowledge is available and can be used for prior elicitation. The time-varying randomisation probabilities are updated based on data accumulated over the course of the trial and depend on two hyper-parameters designed to (i) modulate the allocation imbalance across active arms (T1 and T2) and (ii) preserve the overall allocation of the control arm (C) in order to guarantee a minimum number of patients assigned to this arm. Details on the hyper-parameters and the minimum number of participants in the control arm C have been determined before data collection and are recorded internally. These will remain constant during the study and will be disclosed at the end of the trial to prevent predictability of the algorithm’s allocations.

Accounting for the rare condition and the stratification approach, such a design represents a promising strategy for achieving a near-optimal balance between patient benefit within the study and ensuring statistical guarantees in a small population. By ensuring a minimum sample size for the control arm, when compared to a fixed equal allocation design, such an adaptive design has the potential to enhance the power of the trial, when a single superior arm exists. This is illustrated in Table 4.

Overall, the flexibility of this design results in a potentially more efficient trial framework by increasing the probability of enrolment to arms that show early evidence of efficacy, making efficient use of a small population while giving patients a higher chance of being allocated to the current most efficacious arm of the trial.

### Response definition (adaptation endpoint) for the adaptive algorithm

From animal work, an increase of 30% in BMPR2 is sufficient to rescue signalling and reverse PAH. This is a similar effect size translated to functional and haemodynamic responses in patients in the established PAH clinical trial literature. Therefore, this is the effect considered relevant to inform the potential superiority of an active arm during the ongoing trial. Motivated by this, we define the adaptation endpoint as the binary variable assuming value 1 if the arm shows a change of at least 30% in the primary endpoint, and 0 otherwise. We emphasise that the (binary) adaptation endpoint is computed during interim analyses only; it is meant to guide the response-adaptive design and to determine the adaptive allocation probabilities during the ongoing trial. At the interim analysis stages, no hypothesis testing is performed. The latter is part of the final analyses and it is based on a continuous version of the outcome as documented in “Sample size {14}” section.

## Methods: participants, interventions and outcomes

### Study setting {9}

This is a multicentre trial involving a minimum of 8 UK sites. More sites will be opened, if necessary, in order to achieve the recruitment target. Patients will be recruited over a period of approximately 3 years.

### Eligibility criteria {10}

#### Inclusion criteria

All of the following criteria should be met:

- Aged between 18 and 75 years inclusive
- Weighing 40.0 kg or above at the screening/baseline visit
- Having a diagnosis of group 1 PAH due to the following: idiopathic or heritable PAH with a known mutation in BMPR2
- Being stable on an unchanged PAH therapeutic regime for at least 1 month prior to screening
- Female subjects who are sexually active and of childbearing potential must agree to use two reliable methods of contraception from the beginning of the study (screening/baseline visit) until at least 3 months after the last dose of the investigational product. The method of contraception must be unchanged throughout the study
- Being competent to understand the information given in Independent Ethics Committee (IEC) approved Informed Consent Form and must sign the form prior to the initiation of any study procedures

#### Exclusion criteria

Subjects meeting any of the following criteria must not be enrolled in the study:

- Patients on tumour necrosis factor (TNF) antagonists or other immune targeting biological treatments
- Subject with a known hypersensitivity to the investigational products, the metabolites or formulation excipients
- Subject with severe renal impairment (creatinine clearance < 30 ml/min) at the screening visit
- Subject currently on either an active treatment arm (glycerol phenylbutyrate and hydroxychloroquine) or the trial medication drug class
- Subject with any of the following medical history or current medical conditions: active infection at the time of screening, known hepatitis B or tuberculosis, severe hepatic impairment, alanine aminotransferase or aspartate transferase > 5 × upper limit of normal
- Subject with haematologic and bleeding disorders
- Subject who has had an acute myocardial infarction within the last 90 days prior to screening
- Subject with cardiovascular, liver, renal, haematologic, gastrointestinal, immunologic, endocrine, metabolic or central nervous system disease that, in the opinion of the investigator, may adversely affect the safety of the subject and/or efficacy of the investigational product or severely limit the lifespan of the subject other than the condition being studied
- Subject with a history of malignancies within the past 5 years, except for a subject with localised, non-metastatic basal cell carcinoma of the skin, in situ carcinoma of the cervix or prostate cancer who is not currently or expected, during the study, to undergo radiation therapy, chemotherapy and/or surgical intervention, or to initiate hormonal treatment
- Patient with a history of known retinal disease
- Patient currently taking any of the following contraindicated medications: chloroquine, halofantrine, amiodarone, moxifloxacin, cyclosporin, mefloquine, praziquantel, prochlorperazine, fluconazole, penicillamine and ivabradine
- Female subject who is pregnant or breastfeeding
- Subject who has demonstrated noncompliance with previous medical regimens
- Subject with a recent (within 1 year) history of abusing alcohol or illicit drugs
- Subject who has participated in a clinical study involving another investigational drug or device within 4 weeks before the screening visit

Patients meeting all the eligibility criteria will be provided with the patient information sheet to allow an informed decision regarding their participation, in accordance with relevant regulatory guidelines. Note that assessments conducted as a standard of care do not require informed consent and may be provided as screening data. Where recruitment into another trial is considered to be appropriate and without having any detrimental effect on the StratosPHere 2 trial, co-enrolment is permissible. These should be discussed with the investigators so that the detrimental effect can be explored and agreed present/absent and recorded in the appropriate case report form (CRF).

### Who will take informed consent? {26a}

It is the responsibility of the investigator or a trained delegate to obtain written informed consent from each participant prior to performing any study-related procedures. This will occur after eligibility has been confirmed, the patient has read the patient information sheet and all questions from the patient have been answered. Investigators must ensure that the trial is adequately explained—including the aims, trial treatment, anticipated benefits and potential hazards of taking part in the trial. The PI must also stress that the patient is completely free to refuse to take part in, or withdraw from, the trial at any time.

### Additional consent provisions for collection and use of participant data and biological specimens {26b}

As part of the informed consent process, there is an optional point where participants will give consent for samples and anonymised data to be used in future research studies.

## Interventions

### Explanation for the choice of comparators {6b}

Current evidence from preclinical models suggests two repurposed therapies for achieving functional rescue of specific types of mutations, namely hydroxychloroquine and phenylbutyrate in the treatment of haploinsufficiency and missense mutations, respectively. The two therapies, in addition to standard of care (SoC), will be evaluated in each stratum in comparison with the control treatment (placebo + SoC). As the active treatments have two different administration modes (tablet or liquid), to preserve a level of blinding of the trial, the control treatment is formed by two distinct placebos, each matching the description of the two distinct interventions.

### Intervention description {11a}

- T1: Active arm 1 hydroxychloroquine + SoC. Hydroxychloroquine sulphate (film-coated tablets) is a derivative of chloroquine that inhibits lysosomal degradation and has both antimalarial and anti-inflammatory activities and is now most often used as an immunomodulatory agent in systemic lupus erythematosus and rheumatoid arthritis.
- T2: Active arm 2 glycerol phenylbutyrate + SoC. Phenylbutyrate is a nitrogen-binding medicinal product. It is an odourless, colourless liquid triglyceride containing 3 molecules of glycerol phenylbutyrate linked to a glycerol backbone phenylbutyrate. It is typically used in the management of urea cycle disorders in adults and children.
- C: Control arm either placebo tablets or liquid + SoC. To preserve a level of blinding, the placebo will have two modes of administration matching that of the two active treatments, i.e. one matching tablets (C1) and another one being a liquid (C2).

For both glycerol phenylbutyrate and hydroxychloroquine, there are well-established human data on dosing, and we have adopted established tolerable dose strategies; see Table 1 for more details. All treatments will be administered daily on top of the standard of care.

### Criteria for discontinuing or modifying allocated interventions {11b}

Possible reasons for subject discontinuation include, but are not limited to, the following:

- Development of an adverse event (AE) where continuation of the subject’s participation in the study is thought by the investigator to be inappropriate
- Subject meets blood parameter predefined safety liver stopping criteria
- Subject begins treatment with a prohibited concomitant therapy
- Subject adjudged by the investigator to have a clinically significant neutropaenia or thrombocytopaenia or subject has peripheral blood platelets < 100 × 109/L; subject has a neutrophil count < 2.0 × 109/L on 2 separate tests 24 h apart
- Lost to follow-up, including death

Possible reasons for subject withdrawal from the study include, but are not limited to, the following:

- Pregnancy
- Subject withdraws consent/subject requests to discontinue for any reason
- Subject noncompliance (e.g. refusal or failure to complete study procedures)
- Discretion of the investigator
- Discontinuation of the study at the request of the sponsor, a regulatory agency or an IEC

Treatment specific withdrawal:

- Subjects who develop a pigmentary abnormality, visual field defect or other abnormalities
- Subjects should stop taking the drug immediately if any disturbances of vision are noted, including abnormal colour vision

### Strategies to improve adherence to interventions {11c}

All study staff, both clinical and non-clinical, will receive protocol training before being entered into the delegation and training logs to ensure protocol adherence.

### Relevant concomitant care permitted or prohibited during the trial {11d}

All concomitant medications taken by the participant at the time of enrolment into the study must be recorded in the eCRF. Participants will be asked what medication they are currently taking at each study visit and telephone call. The following medications are to be considered carefully:

- Increased plasma digoxin levels: serum digoxin levels should be monitored in accordance with local trust policies in patients receiving combined therapy
- Antacids may reduce absorption of hydroxychloroquine so it is advised that a 4-h interval be observed between study drug and antacid dosaging
- Hydroxychloroquine may enhance the effects of a hypoglycaemic treatment, a decrease in doses of insulin or antidiabetic drugs may be required
- There may be an association with glycerol phenylbutyrate with increased risk of medicinal product interactions with lipase contained in pancreatic enzyme replacement therapies
- If participants are prescribed short-term (up to 2 week) courses of the following treatments, tablet (but not liquid) trial medication should be withheld for the duration of treatment due to possible interactions with hydroxychloroquine: azithromycin, clarithromycin, erythromycin, ciprofloxacin, remdesivir, fingolimod and laronidase

### Provisions for post-trial care {30}

Study participants will complete the study safety follow-up at 20 weeks post-trial treatment administration, with serious adverse events being recorded up to this time point. At this visit, participation in the study will be complete for all participants.

When withdrawal of patients is due to safety grounds, they will be reviewed locally and patient withdrawal will be at the discretion of the treating team and local PI. Any significant adverse results must be reported to the Data Monitoring Committee via the main study team.

### Outcomes {12}

#### Definition of BMPR2 target engagement primary biomarker endpoint

The primary outcome is a measure of target engagement of the BMPR2 pathway defined by the change in peripheral blood based BMPR2 function, denoted by $\Delta BMPR2$, from baseline (study entry) to 8-week follow-up (i.e. 8 weeks from treatment initiation). The target engagement biomarker is a novel composite panel, defined by a combination of validated measurements of BMPR2 target genes using quantitative PCR (qPCR). Specifically, the panel is an equally weighted mean of the changes (denoted by Δ) in the gene expression of 8 biomarkers evaluated in the StratosPHere 1 trial: *ID3*, *SMAD1*, *SMAD5*, *NOTCH1*, *NOTCH2*, *ID2*, *ARL4C* and *PTGS2* (see Table 2). The primary endpoint is defined for each mutation stratum $s=A, B$, in terms of a change from baseline ($0$ = baseline, or study entry) to follow-up *F* as follows:

$${{\Delta BMPR2}^{s}}_{F}:=\frac{{{|\Delta ID3}^{s}}_{F}|+|{{\Delta SMAD1}^{s}}_{F}|+|{{\Delta SMAD5}^{s}}_{F}|+{{|\Delta NOTCH1}^{s}}_{F}|+{{|\Delta NOTCH2}^{s}}_{F}|+{{|\Delta ID2}^{s}}_{F}|+|{{\Delta ARL4C}^{s}}_{F}|+|{{\Delta PTGS2}^{s}}_{F}|}{8},$$

where:

- With $s=A, B$, we denote the mutation stratum ($A$ = “missense”, $B$ = “haploinsufficiency”).
- With $F$ we indicate the follow-up; for the primary analysis we take $F$ = 8 weeks from treatment initiation.
- With notation ${{\Delta Y}^{s}}_{F}$, where $Y$ denotes the gene expression of biomarker $Y=ID3, SMAD1, SMAD5, NOTCH1, NOTCH2, ID2, ARL4C, PTGS2$, we refer to the change in their gene expression from $0$ = baseline to follow-up $F$. This is defined based on their relative fold change expression ${2}^{-\left|\Delta \Delta Ct{{(Y}^{s}}_{F})\right|}$, following the $\Delta \Delta Ct$ method. *Ct* stands for the cycle threshold (Ct) of the sample as determined by the qPCR methodology.

**Table 2:** Gene names and description

Gene name	Description
***ARL4c***	ADP ribosylation factor like GTPase 4c
***ID2***	Inhibitor of DNA binding 2
***ID3***	Inhibitor of DNA binding 3
***NOTCH 1***	Notch receptor 1
***NOTCH 2***	Notch receptor 2
***PTGS2***	Prostaglandin-endoperoxide synthase 2
***SMAD 1***	SMAD family member 1
***SMAD 5***	SMAD family member 5

Note that we focus on the absolute value of changes Δ (using the module operator | |), disregarding directionalities at this stage. Specifically, taking a generic qPCR gene ${Y}^{s}$ that could be any of the 8 gene products, we define ${{\Delta Y}^{s}}_{F}$ as:

$$\begin{array}{cc}{{\Delta Y}^{s}}_{F}:=\left|{2}^{-\left|{\Delta \Delta Ct{(Y}^{s})}_{F}\right|}-1\right|=\left|{2}^{-\left|\Delta Ct({{ Y}^{s}}_{F})-\Delta Ct({{ Y}^{s}}_{0})\right|}-1\right|,& s=A, B\end{array}$$

where $\Delta Ct({{Y}^{s}}_{t})$, for $t=0, F$, is defined in accordance with the $\Delta \Delta Ct$ method as:

$$\begin{array}{cc}\Delta Ct\left({{Y}^{s}}_{t}\right)=Ct\left({{Y}^{s}}_{t}\right)-Ct\left({{HK}^{s}}_{t}\right),& t=0, F; s=A, B.\end{array}$$

Here, $HK$ denotes the housekeeping gene, and the $\Delta Ct$ is taken to essentially normalise the gene of interest $Y$ to a gene which is not affected by the experiment, hence the housekeeping $HK$ gene term. To improve the stability of the $HK$ gene value, we take it as an average of 4 independent genes.

Note that in the above formulation for ${{\Delta Y}^{s}}_{F}$, we also subtracted 1 (a value indicating no fold change, 1 = ${2}^{-\left|0\right|}$) to be able to interpret the fold change in a traditional scale, e.g. percentage change, and perform a simple average.

#### Safety and exploratory secondary outcomes

- Safety as defined by the incidence and severity of adverse events
- Demonstration of efficacy as defined by change in the following functional, efficacy and quality of life measures from baseline to 16-week follow-up:
  o ${{\Delta BMPR2}^{s}}_{F}$, with change performed from baseline $0$ to follow-up $F=16$ and $F=20$ weeks after treatment initiation, and for each stratum $s=A, B$
  o 6-minute walk test
  o NT-proBNP
  o Health-related quality of life, as measured by EMPHASIS-10
- BMPR2 cell surface protein expression on peripheral blood white cells
- Change in the expression of each individual qPCR biomarker in the primary endpoint panel
- Change in RNAseq peripheral blood expression
- Alternative qPCR Ct normalisation method as given by using the standard curve method

### Participant timeline {13}

All trial-related procedures and interventions will be performed according to the predefined schedule of events in Table 3. Before any study-related procedure, written informed consent will be collected unless that procedure was performed as part of the patient’s standard medical care and is eligible for trial enrolment. After entering the study and being randomised to a treatment arm, participants will be expected to adhere to the assigned arm for 16 weeks with a safety follow-up visit scheduled at week 20 and a follow-up phone call 4 weeks later (week 24). This follow-up time point allows the collection of secondary endpoints, including the BMPR2 panel as well as safety data and longer-term efficacy outcomes to inform phase 2b trials.

**Table 3:** Schedule of events (SoE)

Period of study	Telephone screening	Screening	Treatment schedule					Safety follow-up	Telephone follow-up	Premature study termination
Visit name	Screening 1 (pre-screen)	Screening 2/baseline**	Week 2	Week 4	Week 6	Week 8**	Week 16 EOT**	Week 20 safety follow-up**	Week 24 final follow-up	Premature termination**
Time interval of visit		Day 1	Day 14 (± 3 days)	Day 28 (± 3 days)	Day 42 (± 3 days)	Day 56 (± 7 days)	Day 112 (± 7 days)	30 days after drug discontinuation (± 7 days)	Day 168 (± 7 days)	Visit within 7 days of premature termination
Telephone assessment	X		X	X	X				X	
Mutation carrier/stratum		X								
Inclusion/exclusion criteria		X								
Informed consent		X								
Medical history		X								
Demographics		X								
Urine pregnancy test		X				X	X	X		X
WHO class		X				X	X	X		X
Vital signs		X				X	X	X		X
6MWT		X				X	X	X		X
Disease-specific QOL questionnaire (EMPHASIS-10)		X				X	X	X		
12-lead ECG		X				X	X			X
Randomisation		X				X*				
IMP dispensing		X								
Daily dosing diary		X	X	X	X	X				
AEs/SAEs		X	X	X	X	X	X	X	X	X
Conmeds		X	X	X	X	X	X	X	X	X
ProBNP		X				X	X	X		X
Routine labs		X				X	X	X		X
Drug accountability						X	X			X
Research bloods		X				X	X	X		X

^**^On site visits

^*^Dose recalculated based on height/weight measured at week 8 visit

### Sample size {14}

The trial’s primary objective is to test the hypotheses that BMPR2 modulation (i.e. a mean $E(.)$ increase in our panel measure of BMPR2 target denoted by $E\left({{\Delta BMPR2}^{s}}_{F}\right)>0$; see “Outcomes {12}” section) can be achieved in vivo in a population of patients with mutations in the BMPR2 protein with one of the following therapies: hydroxychloroquine + standard of care (T1) and phenylbutyrate + standard of care (T2) and at *F* = 8-week follow-up. As the two repurposed treatments have distinct mechanisms of action pertinent to strata of BMPR2 mutations, two primary hypotheses will be tested for each stratum *s* independently, with the null ${{H}^{s}}_{0}$ and the alternative ${{H}^{s}}_{1}$ hypotheses defined as:

$$\begin{array}{cc}{{H}^{s}}_{0}=E({{\Delta BMPR2}^{s}}_{F}{)}_{k}-E({{\Delta BMPR2}^{s}}_{F}{)}_{C}\le 0,& s=A, B;k=T1, T2,\end{array}$$

$$\begin{array}{cc}{{H}^{s}}_{1}=E({{\Delta BMPR2}^{s}}_{F}{)}_{k}-E({{\Delta BMPR2}^{s}}_{F}{)}_{C}>0,& s=A, B;k=T1, T2,\end{array}$$

where:

- $E(.)$ denotes the expectation operator.
- $s=A, B$ is the mutation stratum ($A$ = “missense”, $B$ = “haploinsufficiency”).
- $F$ is the follow-up; for the primary analysis, we take $F$ = 8 weeks from treatment initiation.
- *k* denotes the treatment arm, with T1 and T2 being the two active arms and C being the control arm.
- ${{\Delta BMPR2}^{s}}_{F}$ is the primary outcome as defined in (see “Outcomes {12}” section).

We emphasise that this set of overall four hypotheses (which we denote as case II) allows for the possibility of testing on the two experimental arms comparisons for each stratum and was considered for the purpose of quantifying the maximum overall type-I error under a null hypothesis in which none of the treatments works in the stratum *s* ($${{H}^{s}}_{0}).$$ However, the expectation is that only one of these two treatments, if any, works in each mutation stratum, in which case the adaptive design will most likely skew allocation towards the superior treatment to be tested at the end of the study. Thus, under the alternative hypothesis, we expect to compare one single treatment (the one considered to be superior at stage 3, likely T1 in stratum *s* = $$B$$ (“haploinsufficiency”) and T2 in stratum *s* = $$A$$ (“missense”) versus the control arm in each stratum). We denote this most likely scenario as case I, with its set of two hypotheses given by:

$$\begin{array}{cc}{{H}_{0}}^{B}=E({{\Delta BMPR2}^{B}}_{F}{)}_{T1}-E({{\Delta BMPR2}^{B}}_{F}{)}_{C}\le 0,& \text{Stratum }B=\mathrm{"haploinsufficiency"},\end{array}$$

$$\begin{array}{cc}{{H}_{0}}^{A}=E({{\Delta BMPR2}^{A}}_{F}{)}_{T2}-E({{\Delta BMPR2}^{A}}_{F}{)}_{C}\le 0,& \text{Stratum }A=\mathrm{"missense"}.\end{array}$$

The null hypotheses will be tested with an intention to treat approach on an estimated population of 20 patients per each mutation stratum. Notice that 40 patients, 20 per mutation class, is a feasible recruitment envelope based on the realistic patient accrual during the proposed study period given the rare nature of the disease. The testing procedure will use a one-sided non-parametric Wilcoxon test, performed on the continuous primary outcome as defined in Sect. 12, and according to the aforementioned hypotheses, under a frequentist setting.

**Fig. 1:**
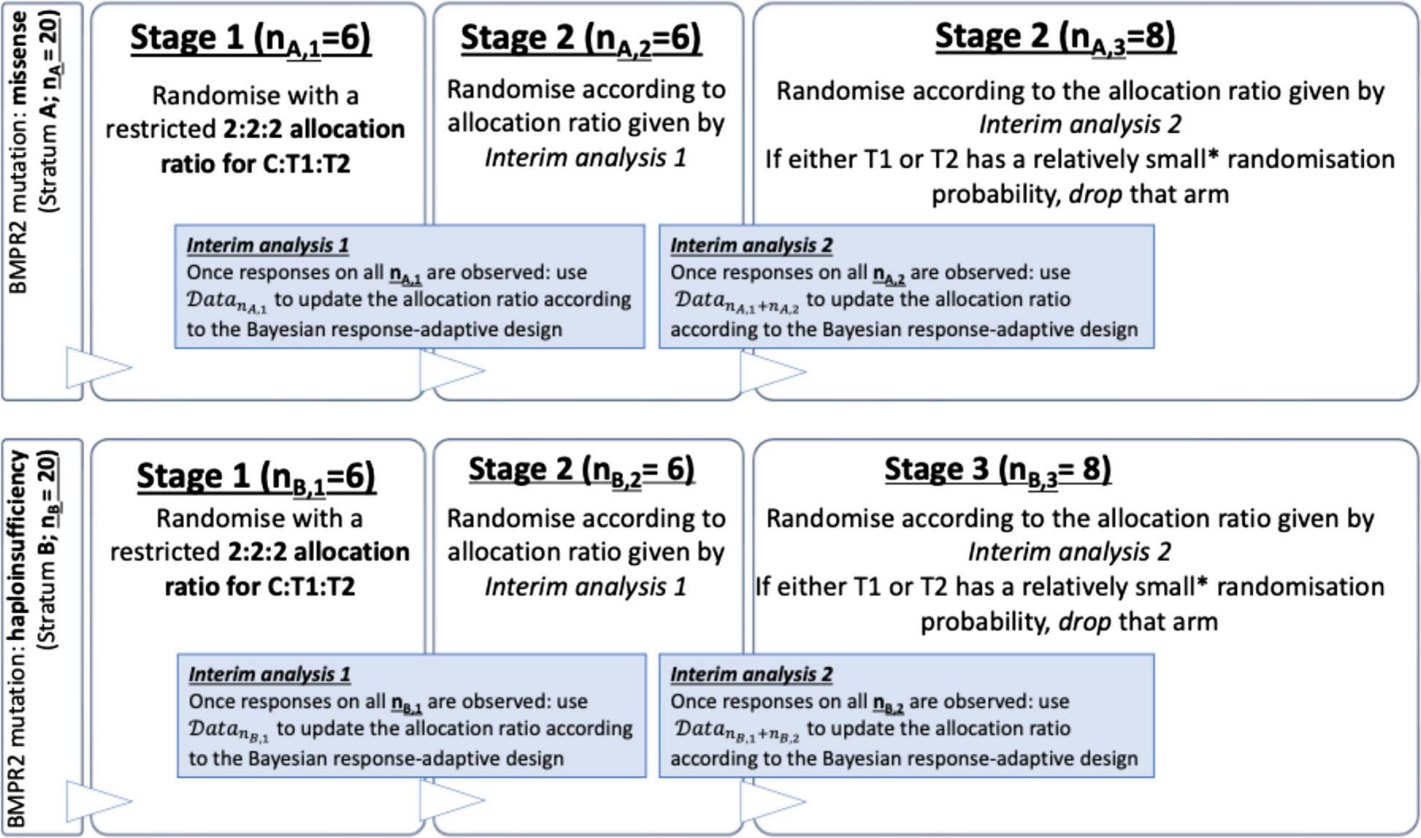
Schematic of the StratosPHere 2 trial design. A = “missense” and B = “haploinsufficiency” denote the two mutation strata; *n* denotes the sample size, with *n*_A,1_ being the sample size of the mutation stratum A at stage 1. T1, T2 and C denote the two active arms and the control arm, respectively. *The probability threshold for dropping an active arm (either T1 or T2, but not C) will be detailed internally and disclosed at the end of the study to ensure PIs do not predict the algorithm’s allocations

Sample size evaluation was carried out by simulations, based on 10,000 replications of the proposed design (as illustrated in Fig. 1). A completely non-parametric approach was adopted for sample size evaluation, both in terms of the statistical test for final analysis (Wilcoxon test) as well as for the data-generating process during the trial replicas (re-sampling from real data points). Specifically, rather than simulating the endpoint based on a modelling assumption, this was generated according to a resampling method that consists of drawing repeated samples from the data samples in the MRC-funded StratosPHere 1 study (Jones RJ, De Bie EM.D.D., Ng AYK, Dunmore BJ, Deliu N, Graf S, Lawrie A, Newman J, Polwarth G, Rhodes C, Hemnes A, West J, Villar SS, Upton PD, UK National Cohort Study of Idiopathic and Heritable PAH Consortium, the Uniphy Clinical Trials Network and Toshner MR: BMPR-II biomarkers for testing therapeutic efficacy in pulmonary arterial hypertension – novel findings from the StratosPHere 1 study, 2024+, under review).

We evaluated the operating characteristics of type-I error and power as well as the probability of patients receiving a superior arm when this exists (i.e. under an expected effect size of 30%, i.e. an overall change of 30% on our primary endpoint, that forms our alternative ${H}_{1}$ hypothesis), in both case I and case II.

As illustrated in Table 4, satisfactory simulation results are expected with 20 patients per stratum. If the design will result in dropping one arm, suggesting the superiority of one of the two active arms (case I), a type-I error control at 10% is expected with an 80% power. However, in case no arm is dropped, with both active arms being equally sampled by the design (case II), we expect the overall type-I error to be higher (19%). Note that this type-I error inflation is due to multiple testing in each stratum and applies to the fixed-balanced design as well.

**Table 4:** Expected operating characteristics (type-I error and power) attainable with a one-sided Wilcoxon test in the system of hypotheses defined for case I (one hypothesis only to be tested for each stratum on the selected arm; overall two hypotheses) and case II (two hypotheses to be tested for each stratum on both experimental arms; overall four hypotheses). Design characteristics are reported by average sample size per arm under the Bayesian response-adaptive design proposed for StratosPHere 2 and a fixed balanced randomised design. All results are based on 10,000 replicas of the trial. The significance level is set to *α* = 12.7% to meet a 10% error control under the adaptive design and the more conservative Wilcoxon test (applied on the continuous endpoint)

Design	Expected type-i error (case I: two ${H}_{0}$)	Expected type-i error (case II: four ${H}_{0}$)	Expected power (under ${H}_{1}$)	Expected allocation of placebo (under ${H}_{1}$)	Expected allocation of superior arm (under ${H}_{1}$)	Expected allocation of inferior arm (under ${H}_{1}$)
Bayesian response-adaptive design	0.10	0.19	0.80	34.0%	47.5%	18.5%
Fixed balanced randomised design	0.11	0.19	0.76	33.3%	33.3%	33.4%

Finally, Table 4 also highlights the benefits of the adaptively randomised design over a fixed strategy in both allocating the most promising arms (column 5) and achieving an increased power in multi-armed cases (column 3) under the alternative.

All analyses were performed using R Statistical Software (v4.1.2; [16]): specifically, the Bayesian design detailed in Sect. 8 was implemented based on user-defined functions, while the testing approach was performed with the R function wilcox.test() from stats package. A dated full copy of the R codes used for the simulation results reported in this protocol is maintained by the sponsors and documented in their systems. This will be disclosed at the end of the study, along with the StratosPHere 1 data used for sample size evaluations, to allow the replicability of the results. This is to minimise the predictability of the allocation sequence by those who enrol participants or assign interventions, who do not have current access to the R codes. Importantly, this procedure will preserve the integrity of the design.

### Recruitment {15}

We aim to recruit approximately 40 participants (20 per mutation stratum) from nationally designated specialist respiratory centres in the UK. To ensure equitable access for patients from across the country, patients will be approached by their direct clinical care team and study team.

## Assignment of interventions: allocation

### Sequence generation {16a}

The allocation sequences will be generated at each adaptive stage following a Bayesian response-adaptive design directly implemented through our R Software code by an unblinded statistician. A mapping strategy will be implemented to reliably translate the continuous allocation probabilities defined by the Bayesian design into discrete allocation ratios. This operational procedure will be detailed in the SAP.

To avoid the allocation sequence being predicted by those who enrol participants or assign interventions, preserving study integrity, certain details on the adaptive design will be masked and disclosed at the end of the study. All these details will be documented internally and will be shared with the Sealed Envelope.

### Concealment mechanism {16b}

A centralised online computer-generated system (Sealed Envelope) will conceal allocation for the study. The allocation sequence is provided to the research pharmacies at each site via this secure system. Once participants are randomised to a treatment arm, site pharmacies will dispense study treatment from stock pre-labelled by the manufacturer/drug packager. These labels have a tear-off portion which when removed will blind the investigational medical products at the point of dispensing.

The system has restricted access of the allocation which is only assigned to specific personnel, i.e. site pharmacies, unblind statistician. All other study personnel involved in direct study conduct and participants are completely blinded to the randomisation system. It will not be possible for the study team to determine treatment allocation using laboratory data during the trial period.

### Implementation {16c}

The allocation sequence will be generated by an unblinded statistician and then provided to Sealed Envelope. An online secure randomisation system provided by Sealed Envelope will assign each participant to the arm they should be allocated to, depending on their mutation subclass. Sealed Envelope will provide an online secure software application for randomising participants into the study to either placebo-based arms (C1/C2) or active arms (T1/T2). The randomisation is stratified by mutation stratum only, and not by centre, which would be unfeasible given the small sample size and the large number of centres that will take part. If a patient discontinues participating in the study, their randomisation code will not be reused, and the patient will not be allowed to re-enter the study. The same system will be used for the whole duration of the trial.

## Assignment of interventions: blinding

### Who will be blinded {17a}

This is a trial with a double placebo presentation of the control group to ensure a level of blinding involving participants, investigators, outcome assessors and study teams. In fact, due to the different modes of administration of the two active treatments, to minimise the possibility of revealing a patient’s assigned treatment group and preserve the blinding of this trial, the control group is formed by two distinct placebos. The physical appearance of the active treatments is matched and these are presented in identical packaging. Study medication will be given a unique code which will be assigned to the subject via the online Sealed Envelope randomisation system. It will not be possible for the study team, including outcome assessors, to determine treatment allocation using the laboratory data during the trial period.

The trial pharmacist at each site will be unblinded. The site can also opt to have an unblinded member of the research team if needed for logistical reasons, but this individual should not be involved in any other trial processes or be part of the direct clinical care team. There will be a blinded study monitor and an unblinded study monitor who will conduct remote monitoring of the site pharmacies.

There is an additional level of blinding to ensure the integrity of this study, which is required due to its response-adaptive nature. For this purpose, the two trial statisticians will remain blinded during the interim analysis and a third unblinded statistician will be instructed to run minimal analysis to update randomisation ratios and will be responsible for generating the allocation sequence. The third statistician will also be unblinded for the purpose of safety data reporting for Independent Data Monitoring Committee (iDMC) meetings.

### Procedure for unblinding if needed {17b}

In the event of a valid medical or safety reason, a request to break the treatment code can be made to the on-call pulmonary hypertension consultant team at the sponsor organisation. Investigators should note that the occurrence of an SAE should not routinely precipitate immediate unblinding. Unblinding will be necessary for SUSAR reporting. If unblinding occurs, the trial medication randomised at baseline (T1, C1 or T2, C2) must be discontinued.

The online Sealed Envelope randomisation system will be used for emergency unblinding. Appropriately trained and delegated on-call pulmonary hypertension consultants at the sponsor organisation will be given the necessary access rights and permission to access this facility. It is the responsibility of the on-call pulmonary hypertension consultant who performed code break to promptly document and communicate the unblinding to the sponsor. For safety monitoring during trial, unblinded results will be forwarded to the iDMC who will address safety issues.

## Data collection and management

### Plans for assessment and collection of outcomes {18a}

The Papworth Trials Unit Collaboration (PTUC) Data Management team will provide data management oversight for the study and will coordinate with the statistical team to ensure that all study data is ready for analysis. Data will be collected by the sites using case report forms designed in collaboration with the trial statistician and trial data manager to ensure that all variables are accurately recorded. Data will be then transcribed on to a trial specific database on the data management system, OpenClinica, with blinded and unblinded access.

#### Baseline

Baseline data will be collected following consent and will include medical history (e.g. PAH diagnosis, BMPR2 mutation and PAH medication), demographics, vital signs, urine pregnancy test, WHO class, blood tests and eligibility confirmation. Once eligibility is confirmed, the following activities will be performed: 6-min walk test (6MWT) functional assessment, 12-lead ECG, blood tests for NT-proBNP and research bloods analysis and disease-specific QOL questionnaire (EMPHASIS-10).

#### Primary outcome data collection

Research bloods will be collected on-site at week 8 (day 56 ± 7 days), week 16 (day 112 ± 7 days) and at week 20 (30 days after drug discontinuation + 7 days). Week 20 will be the last on-site visit. Data collected will be used to determine if any of the two repurposed treatments can achieve target engagement in the two PAH strata of interest, where target engagement will be quantified in terms of a combination of the individual changes in 8 qPCR genes from baseline (prior to treatment initiation) to follow-up (8 weeks from treatment initiation) in the primary endpoint independently for each of the two mutation strata and for each of the treatments considered.

#### Safety and exploratory secondary outcomes

Safety as defined by the incidence and severity of adverse events will be based on the data collected in the AE and SAE CRFs which will get completed as and when an adverse event is identified.

Cardiac functional assessment (NT-proBNP) will be analysed from the routine haematology lab tests collected at baseline and the 8-, 16- and 20-week follow-up and also in cases of premature study termination.

Six-minute walk test data and health-related quality of life, as measured by disease specific QOL questionnaire (EMPHASIS-10), will be collected at baseline and the 8-, 16- and 20-week follow-up and in cases of premature study termination (only 6MWT).

BMPR2 cell surface protein expression on peripheral blood white cells, change in the expression of each individual qPCR biomarker in the primary endpoint panel and change in whole transcriptome using RNAseq of peripheral blood expression will be calculated from the research bloods data collected at baseline and the 8-, 16- and 20-week follow-up and in cases of premature study termination.

Health-related quality of life, as measured by disease specific QOL questionnaire (EMPHASIS-10), will get collected at baseline and the 8-, 16- and 20-week follow-up and in cases of premature study termination.

### Plans to promote participant retention and complete follow-up {18b}

Once participants are enrolled in the study, sites will make reasonable efforts to maintain scheduled visits and follow-up, for the entirety of the study. Study site staff are responsible for developing and implementing local standard operating procedures to achieve reduced rate of participants lost to follow-up.

If participants decide to terminate early from the study or when participants are withdrawn for any reason, they will be asked to complete one last on-site visit within 7 days of premature termination. Participants will return all used and unopened study drug packets. The study procedures at this visit are listed within “Participant timeline {13}” section, schedule of events.

### Data management {19}

Data will be kept on the data management database system, OpenClinica, which is a validated system, tested according to a robust quality system to ensure that it works as designed and is compliant with ICH-GCP and GDPR. Their servers are hosted on the AWS in UK data centres and data encryption is maintained in the database as well as during transit.

The trial specific database will be designed keeping in mind the interim analysis and the adaptive nature of the study. A robust audit trail within OpenClinica tracks all changes to the data and retains a history for each variable, including old and new value, date and time of the change and which user made it.

### Confidentiality {27}

All investigators and trial site staff involved in this trial must comply with the requirements of the Data Protection Act 2018, GDPR and Trust Policy with regard to the collection, storage, processing and disclosure of personal information and will uphold the Act’s core principles. All data used in the formulation of reports to investigators, the sponsor, the funder or ethics will only contain anonymised data. Patient identifiable information will be stored until the end of the study as per relevant jurisdictions, e.g. 15 years in the UK.

### Plans for collection, laboratory evaluation and storage of biological specimens for genetic or molecular analysis in this trial/future use {33}

For trial-related specimen collection, processing and storage see the latest version of the study laboratory manual. Briefly, mononuclear cells will be isolated from whole blood and frozen at − 80 °C and then transferred to liquid nitrogen at the main site. RNA, serum and plasma will be isolated, stored and frozen at − 80 °C. If participants have consented for their samples to be held for future use, they will be held for a period no longer than outlined in the up-to-date regulatory approvals and in accordance with documented consent.

## Statistical methods

### Statistical methods for primary and secondary outcomes {20a}

The data will be analysed following a pre-specified statistical analysis plan (SAP) that will be published in advance of the first interim analysis. The primary outcome, measured at 8 weeks from treatment initiation, will be analysed at the interim time points and used for the final analyses at the end of the study. We will report the mean values, the average variation from baseline to follow-up and 95% confidence intervals to give a range of plausible effects. The primary hypothesis will be tested at the end of the trial for each stratum independently and using a one-sided non-parametric Wilcoxon’s signed rank test. The test will be performed on the (continuous) primary outcome and based on the significance level used for sample size evaluation to achieve the type-I error rates reported for both case I and case II. Being a phase II trial and a rare disease with an expected sample size of 20 patients per stratum, where each mutation and each active treatment has a different pathway, no multiplicity correction will be used for the primary analysis. However, adjustments for multiplicity (mainly of multiple arms comparisons) will be undertaken for the secondary analysis of evaluating a potential effect of any of the two active treatments in the whole trial population (of an expected size of *N* = 40). Such global null will be tested with the same non-parametric Wilcoxon’s signed rank test performed on the continuous primary outcome, and multiple testing adjustment strategies will be reported in the SAP.

Besides the primary and pooled secondary analysis, no other hypothesis testing will be performed on other secondary objectives. All other analyses will be reported with descriptive statistics as exploratory analyses.

A secondary analysis on the primary endpoint will be performed for longer-term (16 weeks and 24 weeks) follow-up data, and additional hypothesis testing analyses would be done according to a procedure detailed in the SAP to ensure error control. For all other endpoints, the analysis will be exploratory and descriptive and will also be detailed in a pre-planned SAP.

### Interim analyses {21b}

Interim analyses will be performed at two pre-specified time points as illustrated in Fig. 1. For each mutation stratum independently, two interim analyses will be carried out based on the 8-week follow-up data for that stratum only. Interim analyses will take into account the primary outcome and will be performed based on the (binary) adaptation endpoint defined to inform the allocation probabilities at subsequent stages (as illustrated in the “Trial design {8}” section). Interim analyses will be performed on the interim analysis population: all those patients that were randomised and for which the primary (8-week follow-up after treatment initiation) endpoint was observed. To perform each of the four (two for each stratum) interim analyses, 6 patients are expected to be enrolled in each stratum’s block. To perform each of the two (two for each stratum) final primary analyses, we expect 8 patients in each stratum’s final blocks.

The interim analysis will be run by an unblinded statistician to preserve the blinding of the trial’s main statisticians. All procedures to safeguard the integrity of the adaptive trial will be recorded and detailed in the SAP.

The interim analysis will only be performed with the purpose of reporting safety and deciding the allocation ratios, but no additional analysis will be performed at these stages.

### Methods for additional analyses (e.g. subgroup analyses) {20b}

The present study is a phase II precision medicine study that aims to investigate the intervention effects among two subgroups, characterised by two different BMPR2 mutations, of the study population. Thus, the study design involves stratification of the target population into two subgroups based on their mutation class (missense and haploinsufficiency), and the primary final analysis will be performed for each subgroup independently. Note that the two categorical strata are defined by protocol and patients are directly assigned to one of the two groups before randomisation.

Additional subgroup analyses will be all exploratory and only descriptive summaries will be reported; these may potentially involve all the baseline variables reported in Sect. 18a. In case of any subgroup analysis on continuous-type of variables, the groups will be defined upon domain thresholds. A detailed list will be provided in the SAP.

### Methods in analysis to handle protocol non-adherence and any statistical methods to handle missing data {20c}

Intention to treat is the main analysis approach for this study. The primary analysis will be based on an intention to treat population, consisting of all eligible subjects who were randomised, regardless of whether they received the treatment randomly allocated to them or completed follow-up. The data will be analysed assuming that the patient received the treatment they were randomly allocated and as indicated.

In principle, the interim and final analyses will be based on all randomised subjects with the primary endpoint at 8-week follow-up available. From previous studies, we do not expect missing data in the primary endpoint, which is built based on laboratory results. Furthermore, this is a rare condition, and previous studies conducted at the centres of interest show a high participation rate. However, the impact of missing data on both design and analysis performances will be investigated in different potential missing data scenarios. The missing data mechanism will be explored and predetermined imputation techniques, adequately documented in the SAP and justified with simulation studies, may be applied as appropriate. We note that imputation modes may be different for the binary adaptation endpoint used during interim analyses and the continuous primary endpoint for the final analysis.

A “per protocol” analysis will be reported if enough data on non-adherence is available and the extent of bias on the estimates will be discussed at the final analysis stage. The per-protocol population consists of subjects who completed the whole study period (complete cases) without any major protocol deviations. Any subjects who did not receive the treatment randomly allocated to them will be excluded from the per-protocol population. Analyses of this population are seen as a secondary and as a form of sensitivity analysis.

### Plans to give access to the full protocol, participant-level data and statistical code {31c}

Royal Papworth Hospital NHS Foundation Trust, as sponsor and lead site, will manage participant-level data, which can be made available if requested by a member of the public. The statistical analysis code will be stored according to local standard operating procedures and can also be made available upon request to the corresponding author.

## Oversight and monitoring

### Composition of the coordinating centre and trial steering committee {5d}

The role, composition and constitution of the iDMC and Trial Steering Committee (TSC) will follow MRC/UKRI Guidance, Sect. 6.1 [17]. There will be a group running the trial day-to-day and providing organisational support. The TSC will provide oversights and will meet for every interim analysis.

### Composition of the data monitoring committee, its role and reporting structure {21a}

The iDMC will be comprised of an unblinded independent group, as defined in a separate charter document, which will define the role of the iDMC. Additionally, ad hoc iDMC meetings may be triggered. In the event that adaptations to this guidance are required to support effective trial oversight, these will be agreed upon with the funder in advance. Membership of the iDMC and TSC will be specified in a separate document. An iDMC Charter will be agreed upon and signed by appointed members.

### Adverse event reporting and harms {22}

#### Assessment of safety

Assessment of safety will be performed on the safety population, defined as all participants who received any study arm. Participants will receive a telephone call at week 2 (day 14 ± 3 days), 4 (day 28 ± 3 days) and 6 (day 42 ± 3 days) to check concomitant medications and the occurrence of adverse events (AEs). The research team must, in particular, be vigilant for new or worsening signs of cardiomyopathy, hypoglycaemia, visual disturbances and psychiatric reactions especially in the first month of treatment as events have been reported in patients taking hydroxychloroquine with no prior history of psychiatric disorders.

The information used for assessing whether an adverse reaction is expected is contained in the Summary of Product Characteristics (SPC). Hydroxychloroquine may cause abdominal pain and/or nausea. All other expected adverse reactions, AEs or SAEs within this trial are listed in the latest MHRA-approved version of the Reference Safety Information, as specified in Table 5.

**Table 5:** Reference safety section

Name of the investigational medicinal product	SPC date
Hydroxychloroquine sulphate. 200 mg film-coated tablets	Revision of text: 19/01/2022
RAVICTI—glycerol phenylbutyrate. 1.1 g/ml oral liquid	Revision of text: 03/05/2022

All harms will be collected and recorded on the trial eCRF.

#### Toxicity—emergency procedures

PAA, the active metabolite of glycerol phenylbutyrate, is associated with signs and symptoms of neurotoxicity and could accumulate in patients who receive an overdose. In case of overdose, the medicinal product should be discontinued and the patient monitored for any signs or symptoms of adverse reactions.

The symptoms of overdosage/toxicity with hydroxychloroquine may include headache, visual disturbances, cardiovascular collapse, convulsions, and hypokalaemia, rhythm and conduction disorders, including QT prolongation, Torsade de Pointes, ventricular tachycardia and ventricular fibrillation, width-increased QRS complex, bradyarrhythmia, nodal rhythm, atrioventricular block, followed by sudden potentially fatal respiratory and cardiac arrest. Immediate medical attention is required, as these effects may appear shortly after the overdose. Participants should be urgently admitted to hospital and study drug discontinued until assessment by the responsible principal investigator. Procedures for toxicity include evacuation of the stomach, either by emesis or by gastric lavage. Activated charcoal in a dose at least five times of the overdose may inhibit further absorption if introduced into the stomach by tube following lavage and within 30 min of ingestion of the overdose. Consideration should be given to administration of parenteral diazepam in cases of overdosage; it has been shown to be beneficial in reversing chloroquine cardiotoxicity. Respiratory support and shock management should be instituted as necessary. If toxicity with study drug is suspected and confirmed, the unblinding procedure will be triggered and patient withdrawn from the study.

### Frequency and plans for auditing trial conduct {23}

Monitoring will be performed for each site by the sponsor. In the event there are issues with the site in regard to documentation, consent or completion of data in OpenClinica then additional triggered on-site monitoring may be conducted at the discretion of the project management/QA team.

### Plans for communicating important protocol amendments to relevant parties (e.g. trial participants, ethical committees) {25}

All study (including protocol) amendments will be submitted for approval to the relevant ethical and governance committees. Sites will be informed of all approved minor or substantial amendments and will be asked to review and confirm approval at the local site level. Participants will be informed and reconsented if deemed necessary by the sponsor. The study team will also provide protocol training for all protocol amendments.

## Dissemination plans {31a}

Several members of the study team are national experts in clinical research and implementation of new research findings into clinical practice. On completion of the research, final trial report will be prepared and submitted to the MHRA and REC. Once completed and peer reviewed, social, professional and mainstream media will be contacted to inform as many people as possible about the study protocol, statistical analysis plan, findings and recommendations for future clinical practice and health policy. The results of StratosPHere 2 will be made publicly available following funder (MRC) approval. A separate Publication Policy document will be available on request.

## Discussion

To our knowledge, this is the first trial to assess the efficacy of therapies as a BMPR2 modulator in pulmonary hypertension. The trial is designed to efficiently explore precision medicine hypotheses in a small population of patients stratified by subclass mutation. The study is also contributing with the first use of a BMPR2 panel as a primary endpoint candidate to test these classes of hypotheses around the mechanism of the disease rather than the consequences of it. This trial has proposed the use of a novel primary endpoint that is consistent with adapting the allocation ratio in line with patient recruitment.

The choice of a Bayesian response-adaptive design allows for the most efficient use of sample size to address all the challenges of including a concurrent placebo control group and testing a precision medicine hypothesis in a rare disease setting. This design was chosen with consideration to optimally balance the trial’s sample size constraints and to keep a low number of eligible patients randomised to a weakly performing arm while improving the ability to decide whether further study of these therapies in this setting is warranted. Our proposed design, compared with a balanced design with a fixed and equal allocation ratio, shows potential substantial gains in terms of both statistical power for the hypothesis of interest and a higher chance for patients to be allocated to a superior arm (when it exists).

The primary objective of the trial is to show potential target engagement of the BMPR2 pathway in a population of patients with BMPR2 mutations stratified by mutation subtypes using two different approaches to BMPR2 rescue: glycerol phenylbutyrate and hydroxychloroquine. The choice of the PCR biomarker panel as the primary outcome measure allows such interpretation since it is a feasible and reproducible short/medium-term endpoint and allows for the design to adapt in a frequency that matches recruitment in an aligned way. Additional secondary efficacy measures (6MWT, NT-proBNP, EMPHASIS-10 patient-related outcome measure) will inform future later phase studies.

## Trial status

Protocol version number 2.0 dated 15/08/2023. This study has started recruitment on 08/03/2024. The planned end date of the study is November 2026.

## Data Availability

Ownership of the data arising from this trial resides with the study team. The results of StratosPHere 2 will be made publicly available following funder (MRC) approval. A separate Publication Policy document will be reported in SAP and the data supporting the findings of this study will be available from the authors upon request.

## Abbreviations

6MWT: 6-Minute walk test
AE: Adverse event
AR: Adverse reaction
BMPR2: Bone morphogenetic protein type 2 receptor
BNP: Brain natriuretic peptide
CRF: Case report form
Ct: Cycle threshold
eCRF: Electronic case report form
ER: Endoplasmic reticulum
HK: Housekeeping gene
HRA: Health Research Authority
IEC: Independent Ethics Committee
iDMC: Independent Data Monitoring Committee
MHRA: Medicines and Healthcare products Regulatory Agency
NT: N-terminal
NT-proBNP: N-terminal prohormone of brain natriuretic peptide
PAH: Pulmonary arterial hypertension
PTUC: Papworth Trials Unit Collaboration
SAE: Serious adverse event
SAP: Statistical analysis plan
SoC: Standard of care
SPC: Summary of Product Characteristics
TSC: Trial Steering Committee
